# 

**Published:** 1904-03

**Authors:** 


### CONTENTS.

ORIGINAL COMMUNICATIONS—

MASTOIDITIS: CAUSES, PREVENTION AND TREATMENT. By J. M. Crawford, M D., Atlanta, Ga.......... 793
PNEUMONIA. By James B. Baird, M.D., Atlanta, Ga.......... 800
POST-MORTEM FINDINGS IN A CASE OF DIABETES MELLITUS. By Wm. C. Pumpelly, M.D., Pathologist State Sanitarium, Milledgeville, Ga.......... 809
PRELIMINARY REPORT ON THE UTILIZATION OF PLASTIC LYMPH AS A REPARATIVE TISSUE IN ABDOMINAL SURGERY. By E. C Davis, M.D., Atlanta, Ga.......... 814
THE X-RAY TREATMENT OF INOPERABLE CARCINOMA OF THE BREAST; REPORT OF A CASE. By James N. Brawner, M.D, Atlanta, Ga.......... 816

SOCIETY REPORTS—

THE ATLANTA SOCIETY OF MEDICINE.......... 718

CORRESPONDENCE.......... 822

EDITORIAL NOTES AND COMMENTS—

THE DEATH OF DR. VIRGIL O. HARDON.......... 825
NATURAL CLEANLINESS OF PLANTS AND ANIMALS.......... 827
EDITORIAL BREVITIES.......... 829

NEWS AND NOTES.......... 830

CONTENTS CONTINUED.......... X

